# *In vitro* anti-prostate cancer efficacy and phytochemical composition of the dichloromethane and ethyl acetate leaf extracts of *Vitex doniana* (sweet)

**DOI:** 10.3389/fphar.2024.1483856

**Published:** 2024-11-14

**Authors:** Gervason Moriasi, Mathew Ngugi, Peter Mwitari, George Omwenga

**Affiliations:** ^1^ Department of Biochemistry, Microbiology and Biotechnology, School of Pure and Applied Sciences, Kenyatta University, Nairobi, Kenya; ^2^ Department of Medical Biochemistry, School of Medicine, Mount Kenya University, Thika, Kenya; ^3^ Centre for Traditional Medicine and Drug Research, Kenya Medical Research Institute, Nairobi, Kenya

**Keywords:** ethnomedicine, MTT assay, RT-qPCR, Vero-CCL-81, DU-145, natural pruducts, gene expression profile

## Abstract

**Background:**

Prostate cancer is a significant global health concern, particularly among ageing male populations, with a disproportionately higher burden in sub-Saharan Africa. Conventional treatments, though effective, are costly and cause devastating side effects which limit their clinical benefits. Hence, this study evaluated the *in vitro* antiprostate cancer properties and secondary metabolites of dichloromethane and ethyl acetate lead extracts of *Vitex doniana* to explore safer and efficacious natural alternatives based on ethnomedicinal claims.

**Methods:**

Phytochemical profiling was conducted using gas chromatography-mass spectrometry (GC-MS) analysis to identify secondary metabolites in the extracts. The cytotoxic effects of the extracts were determined through the MTT assay using Vero CCL-81 cells and DU-145 cells. The expression profile of the selected genes (*ar, bcl2, caspase-3, cdk1*, and *p53*) in DU-145 cells treated with the study extracts was investigated using RT-qPCR.

**Results:**

GC-MS analysis revealed 10 secondary metabolites in the dichloromethane extract and 27 secondary metabolites in the ethyl acetate extract of *V. doniana* leaves, with the majority being sesquiterpenes, diterpenoids, and phytosterols. The dichloromethane and ethyl acetate leaf extracts of *V. doniana* exhibited low cytotoxicity against normal mammalian epithelial cells (Vero CCL-81), with CC_50_ values of 1,238.85 μg/mL and 964.81 μg/mL, respectively. Besides, the ethyl acetate leaf extract of the studied plant demonstrated potent anti-prostate cancer activity against DU-145 cells, with an IC_50_ of 35.68 μg/mL and a high selectivity index (SI) of 27.04. Likewise, the dichloromethane leaf extract of this plant displayed cytotoxic effects (IC_50_: 287.01 μg/mL) and a selectivity index of 4.32. The reference drug (Doxorubicin) showed a higher toxicity against Vero CCL-81(IC_50_: 0.41 μg/mL) and DU-145 (IC_50_: 0.28 μg/mL) cells and a lower selectivity index of 1.46. The DU-145 cells treated with the studied plant extracts exhibited notable upregulation of *ar* and *bcl2*, and normalization of *caspase 3, cdk1* and *p53* expression.

**Conclusion:**

The studied plant extracts possess *in vitro* anti-prostate cancer properties and could be promising candidates for further preclinical studies aimed at developing novel botanical-based therapies for the management of prostate cancer.

## 1 Introduction

Prostate cancer remains a significant public health challenge, particularly among aging male populations, and its global prevalence continues to rise despite advances in diagnostics and treatments (Menegoz et al., 2019). The burden of cancer is disproportionately high in low- and middle-income countries (LMICs), particularly in sub-Saharan Africa, such as Kenya (Ministry of Health MOH-Kenya, 2021; Sung et al., 2021). This disparity is attributed to a complex interplay of genetic, socioeconomic, and environmental factors (Menegoz et al., 2019). Epidemiological data shows prostate cancer is the leading malignancy among men in Kenya, accounting for approximately 8% (3,582) of new cancer diagnoses and 6.6% (2,029) of cancer-related deaths annually (Ministry of Health MOH-Kenya, 2021; Sung et al., 2021), and is exacerbated by weak healthcare infrastructure, the high cost of cancer treatment, and insufficient access to quality healthcare services (Price et al., 2012; Ocran Mattila et al., 2021). Moreover, despite the advancements in conventional medicine, the current therapeutic interventions for prostate cancer, such as chemotherapy and radiation are costly and often cause severe side effects, limiting their clinical benefits (Ahmed et al., 2018). These limitations underscore the need for alternative therapies that are safe and efficacious to mitigate the existing challenges.

The use of botanical preparations as a complementary or alternative approach to cancer treatment has existed in various cultures since antiquity (Kareru et al., 2007; James et al., 2018; Aumeeruddy and Mahomoodally, 2021). However, despite its widespread application, the integration of these botanical therapies into conventional oncology still faces considerable challenges (Gakuya et al., 2020). For instance, there is a paucity of empirical data to validate the safety and therapeutic potential of traditionally utilized botanical products (Gakuya et al., 2020; George, 2011). Furthermore, many botanical preparations contain metabolites that remain unidentified, heightening the risk of toxicity and adverse interactions with conventional cancer therapies (Nasri and Shirzad, 2013; Wanjiru et al., 2022). This highlights the urgent need for scientific investigations to assess the safety, efficacy, and pharmacological actions of these botanical drugs. Such research is crucial for addressing existing knowledge gaps and facilitating the development of evidence-based strategies to harness the therapeutic potential of botanical drugs in cancer care.

*Vitex doniana* (Sweet), is native Kenyan tree belonging to the Verbenaceae family. It is locally known as “*Muhuru*” (Kikuyu), “*Muekelwet*” (Kipsigis), “*Mfundu*” (Swahili), and “*Jwelu*” (Luo) (Patrick et al., 2005). Its leaves are used traditionally by various communities to treat microbial infections, inflammatory diseases, and allergies, and cancer (Jean et al., 2019; Das et al., 2022). Previous studies have identified a wide range of secondary metabolites, including alkaloids, terpenes, saponins, flavonoids, as well as essential vitamins (A, B1, C), and minerals such as potassium, iron, magnesium, calcium, and zinc (Das et al., 2022; Ifeanacho et al., 2019; Dah-Nouvlessounon et al., 2023). These metabolites likely contribute to the plant’s medicinal properties. Scientific research has shown that *Vitex doniana* exhibits antimicrobial, antioxidant, anti-inflammatory, and hepatoprotective activities (Jean et al., 2019; Das et al., 2022; Kamal et al., 2022). However, despite its widespread usage among Kenyan communities to treat prostate cancer, there is a paucity of empirical information to validate its safety and efficacy.

Therefore, this study aimed to investigate the antioxidative stress, anti-prostate cancer, and phytochemical profile of *V. doniana* to appraise its pharmacological potential. These findings will pave the way for future studies aimed at integrating botanical drugs into clinical applications, offering an integrative approach to prostate cancer management with fewer adverse effects than conventional treatments.

## 2 Materials and methods

### 2.1 Plant material

Mature fresh leaves of *V. doniana* were harvested sparingly from its natural habitat in Mbeere North Sub-County, Embu County, Kenya, according to a procedure outlined by Carter et al. (Carter et al., 2007). This plant was selected based on its ethnomedicinal use by the local community for managing prostate cancer. It was first Identified locally as “*Muburu*,” by an acknowledged traditional herbalist and then authenticated macroscopically and microscopically by a competent taxonomist (Mr. Kennedy Matheka) at the Department of Botany, of the National Museums of Kenya (NMK/BOT/CTX1/4), where duplicate specimens were preserved for future reference. The collected leaves were transported in woven sisal bags to our research laboratory, at Department of Biochemistry, Microbiology, and Biotechnology, Kenyatta University, where they were air-dried for 2 weeks in a well-ventilated room away from direct sunlight. The dried leaves were ground into a powder using an electric mill, carefully packed in labelled khaki envelopes, and stored on a clean dry shelf until required for extraction.

### 2.2 Extraction

A modified cold maceration procedure described previously (Harborne, 1998) and adapted by Moriasi et al. (Moriasi et al., 2021) was used in this study. In brief, two 250-g portions of the plant material were separately soaked in 1,000 mL of analytical grade ethyl acetate and dichloromethane, stirred and shaken intermittently for 48 h. After that, the mixtures were decanted and filtered through Whatman No. 1 filter paper. The ethyl acetate and dichloromethane filtrates were concentrated *in vacuo* at 50°C, and 30°C, respectively, using a rotary evaporator. The percentage yields were calculated according to the method of Truong et al. (Truong et al., 2019), as shown in (Equation 1) and the extracts were stored in brown glass vials awaiting experimentation.

$$\%\text{ Yield}=\frac{\text{weight}\;\text{of}\;\text{the}\;\text{extract}}{\text{weight}\;\text{of}\;\text{the}\;\text{macerated}\;\text{sample}}x\;100$$ (1)

### 2.3 GC-MS analysis

For this study, analysis of the secondary metabolites in the two plant extracts was performed using a Shimadzu QP 2010-SE GC-MS system with an auto sampler connection following a previously established protocol (Ahmad et al., 2023). Analytical-grade dichloromethane and ethyl acetate were sourced from Sigma-Aldrich. The dichloromethane and ethyl acetate leaf extracts of *V. doniana* were dissolved in their respective solvents to obtain a concentration of 1 mg/mL. The prepared extract solutions were filtered through 0.45 µm PTFE syringe filters after which they were injected in split mode at a 10:1 into the GC-MS system. Ultrapure Helium (He) was used as the carrier gas at a linear velocity of 35 cm/s. A BPX5 nonpolar column (30 m × 0.25 mm ID; 0.25 μm film thickness) was used for separation. The GC temperature was programmed as follows: 60°C; 10°C/min to 200°C (hold time1min); 10 ^°^C/min to 280°C (10 min). The total runtime was 33 min. Injection temperature was set to 250°C, and interface temperature was set at 250°C. The EI ion source was set at 200°C, working in electron impact (EI) mode at 70 eV. Mass analysis was done in Scan mode, with a scan range of m/z values of 35–550 a.m.u.

### 2.4 Determination of *in vitro* cytotoxicity and anti-prostate cancer effects

#### 2.4.1 Preparation of plant extracts

Each extract was precisely weighed (100 mg), dissolved in 10 mL of dimethyl sulfoxide, and vortexed to obtain a working stock solution. From this solution, 100 μL was diluted with phosphate-buffered saline to a final volume of 1,000 μL, resulting in a concentration of 1,000 μg/mL. Then, three-fold serial dilution was performed producing seven concentrations: 1.37 μg/mL, 4.12 μg/mL, 12.35 μg/mL, 37.04 μg/mL, 111.11 μg/mL, 333.33 μg/mL, and 1,000.00 μg/mL. Similarly, the reference drug, Doxorubicin, was serially diluted to produce working concentrations of 0.04 μg/mL, 0.12 μg/mL, 0.37 μg/mL, 1.11 μg/mL, 3.33 μg/mL, 10 μg/mL, and 30 μg/mL.

#### 2.4.2 Cell culture and maintenance

The experimental American Type Culture Collection (ATCC) cell lines were retrieved from the tissue culture laboratory hosted at the Centre for Virus Research (CVR) of the Kenya Medical Research Institute (KEMRI), cultured, and maintained according to previously established protocols (Markossian et al., 2021). Vero CCL-81 cells were grown in Eagle’s Minimum Essential Medium (EMEM) enriched with 10% foetal bovine serum (FBS), 2 mM L-glutamine, and an antibiotic-antimycotic mixture (100 units/mL penicillin and 0.1 mg/mL streptomycin), under sterile conditions. Prostate cancer (DU-145) cells were maintained in high glucose Dulbecco’s Modified Eagle’s Medium (DMEM), supplemented with 10% FBS, 2 mM L-glutamine, and 1% antibiotic-antimycotic mixture. All cell lines were incubated in a humidified (65%) chamber with 5% CO_2_ at 37°C for 48 h. Cell growth was monitored three times a week, and upon reaching ≥90% confluence, they were trypsinised, passaged, and resuspended in fresh media for subsequent assays.

#### 2.4.3 The 3-(4,5-dimethylthiazol-2-yl)-2,5-diphenyltetrazolium bromide (MTT) assay

The cytotoxicity of plant extracts on Vero CCL-81 cells was assessed *in vitro* using a modified MTT assay method (Markossian et al., 2021). Briefly, 100 µL of medium was transferred into 96-well plates, where cells were seeded at a density of 2 × 10^4^ cells per well and allowed to attach overnight under specified conditions (Section 2.4.2). Following cell attachment, varying concentrations (0–1,000 μg/mL) of the plant extracts, dissolved in 0.1% dimethyl sulfoxide (DMSO), and the reference drug (doxorubicin) were added to the wells in triplicate, and the plates were incubated for 48 h as described in Section 2.4.2. Subsequently, 10 µL of the MTT reagent (5 mg/mL) was added to each well, and after a 4-h incubation, the supernatant was aspirated and replaced with 100 µL of 0.1% DMSO. The experiment was performed in four replicates. The absorbance of the formazan crystals was measured at 570 nm and used to calculate the percentage cytotoxicity and inhibition of cancer cell proliferation as shown in (Equation 2).

$$\%\;\text{Cytotoxicity}/\text{Inhibition}=1-\left(\frac{\text{The}\;\text{absorbance}\;\text{of}\;\text{treated}\;\text{cells}}{\text{The}\;\text{absorbance}\;\text{of}\;\text{control}\;\text{cells}}\right)\times 100$$ (2)

#### 2.4.4 Determination of the extracts’ cytotoxic and antiproliferative efficacy

The median cytotoxic concentrations (CC_50_) for Vero CCL-81 cells and median inhibitory concentrations (IC_50_) for DU-145 cells were interpolated from a linear regression plot of percentage cytotoxicity/inhibition versus concentration, to appraise the extracts’ safety and efficacy. Additionally, the selectivity indices (SI) for DU-145 cells were calculated using the formula provided in Equation 3.

$$\text{SI}=\left(\frac{{\text{CC}}_{50}\;\text{value}\;\text{for}\;\text{Vero}\;\text{CCL}-81\;\text{cells}}{{\text{IC}}_{50}\;\text{value}\;\text{for}\;\text{cancer}\;\text{cells}}\right)$$ (3)

#### 2.4.5 Determination of the expression profiles of cancer-associated genes in the DU-145 treated with the selected plant extracts

The expression profile of key genes associated with cancer initiation and progression were analysed using quantitative real-time polymerase chain reaction (RT-qPCR). The DU-145 cells (1 × 10^6^) were seeded in 96-well plates and treated with the plant extracts at concentrations corresponding to their IC_50_ values and incubated for 48 h at conditions described in Section 2.4.2. Total RNA was extracted using a commercial total RNA Miniprep Kit (Solis BioDyne), and its concentration and purity were assessed using a Nanodrop spectrophotometer (ThermoFisher Scientific). The RNA (2 µg) was reverse transcribed into complementary DNA (cDNA) using a cDNA synthesis kit (Solis BioDyne). Afterward, RT-qPCR of the cDNA was added SYBR Green dye and the target genes (Bcl_2_, AR, *CDK*1, *p53*, caspase 3) were amplified using specific primers (Table 1) using a QuantStudio™ 5 System (ThermoFisher Scientific). Gene expression levels were analysed using the comparative threshold (CT) method and normalized with *gapdh* and *actb*. The fold changes were calculated using the relative quantification (2^−ΔΔCt^) approach (Rao et al., 2013), and expressed as fold changes (fold changes).

**TABLE 1:** Target genes and their respective primers.

Target gene		Primer sequence	
		Forward [5′-3']	Reverse (3′-5′)
1	*actb*	GCC​AAC​TTG​TCC​TTA​CCC​AGA	AGG​AAC​AGA​GAC​CTG​ACC​CC
2	*p53*	CTT​CGA​GAT​GTT​CCG​AGA​GC	GAC​CAT​GAA​GGC​AGG​ATG​AG
3	*Caspase3*	CAA​AGA​GGA​AGC​ACC​AGA​ACC​C	GGA​CTT​GGG​AAG​CAT​AAG​CGA
4	*cdk1*	GAA​CAC​CAC​TTG​TCC​CTC​TAA​GAT	CTG​CTT​AGT​TCA​GAG​AAA​AGT​GC
5	*bcl2*	GGC​CTC​AGG​GAA​CAG​AAT​GAT	TCC​TGT​TGC​TTT​CGT​TTC​TTT​C
6	*ar*	GCT​TTA​TCA​GGG​AGA​ACA​GCC​T	TGC​AGC​TCT​CTC​GCA​ATC​TG
7	*gapdh*	CCC​CAC​CAC​ACT​GAA​TCT​CC	CTC​ACC​TTG​ACA​CAA​GCC​CA

### 2.5 Data management, statistical analysis, and reporting

Quantitative data, from *in vitro* cytotoxicity and antiproliferative assays were organised using Microsoft Excel (Office 365) before being analysed using GraphPad Prism version 10.2. Descriptive statistics were presented as mean ± standard deviation $\left(\bar{x}\pm SD\right)$ across replicate experiments. For statistical comparisons, unpaired student t-test statistic or one-way ANOVA with Tukey’s *post hoc* test were conducted, at a significance of *P* < 0.001. Gene expression analysis was performed using the 2^−ΔΔCt^ method on the QuantStudio™ 5 System. The metabolites in the extracts were identified by matching their mass spectra and retention indices with the NIST library using a homologous series of n-alkanes (C8-C20) under identical GC-MS conditions. They were further verified by comparing their mass spectra and retention indices with reference standards from PubChem (nih.gov), NIST Chemistry WebBook, and scholarly literature. Metabolite names, class, molecular weight, molecular formula, and retention time were tabulated. The relative abundance of each metabolite was determined by analysing peak areas in the total ion chromatogram.

## 3 Results

### 3.1 Phytochemical compounds identified in the dichloromethane and ethyl acetate leaf extract of *Vitex doniana*

GC-MS analysis of the dichloromethane and ethyl acetate leaf extracts from *V. doniana* revealed a diverse array of metabolites, as detailed in Table 2. The dichloromethane extract contained ten distinct metabolites, predominantly diterpenoids, steroids, phytosterols, and fatty acid esters. γ-Sitosterol was the most prevalent metabolite, accounting for 41.75% of the extract, while Stigmasta-3,5-dien-7-one and Stigmast-4-en-3-one accounted for 21.19% and 10.91%, respectively (Table 2). Conversely, Neophytadiene and Phytol acetate were present in small amounts, at 1.18% and 0.51%, respectively (Table 2).

**TABLE 2:** Secondary metabolites identified in the Dichloromethane (VD-DCM) and Ethyl Acetate (VD-EA) leaf Extracts of *Vitex doniana*.

Extract	Class	Name	Molecular formula	Molecular weight	Retention time (minutes)	% area
VD-DCM	Diterpenoid	Neophytadiene	C_20_H_38_	278	16.719	1.18
	Diterpenoid	Phytol, acetate	C_22_H_42_O_2_	338	19.637	0.51
	Steroid	9,19-Cyclocholestan-3-ol, 14-methyl-, (3β,5α)-	C_28_H_48_O	400	22.919	2.50
	Phytosterol	Stigmasta-4,22-dien-3β-ol	C_29_H_48_O	412	23.638	2.96
	Phytosterol	γ-Sitosterol	C_29_H_50_O	414	25.864	41.75
	Fatty acid ester	6-Fluoro-2-trifluoromethylbenzoic acid, eicosyl ester	C_28_H_44_F_4_O_2_	488	26.020	3.81
	Triterpenoid	9,19-Cyclolanost-24-en-3-ol, (3β)-	C_30_H_50_O	426	27.940	9.17
	Triterpenoid	Lup-20(29)-en-3-one	C_30_H_48_O	424	28.619	6.02
	Phytosterol	Stigmasta-3,5-dien-7-one	C_29_H_46_O	410	29.834	21.19
	Phytosterol	Stigmast-4-en-3-one	C_29_H_48_O	412	31.589	10.91
VD-EA	Sesquiterpenoid	(3R,3aR,3bR,4S,7R,7aR)-4-Isopropyl-3,7-dimethyloctahydro-1H-cyclopenta [1,3] cyclopropa[1,2]benzen-3-ol	C_15_H_26_O	222	11.954	0.99
	Sesquiterpene	α-Copaene	C_15_H_24_	204	12.219	1.53
	Sesquiterpene	Ylangene	C_15_H_24_	204	13.061	0.68
	Sesquiterpene	Bicyclo[4.4.0]dec-1-ene, 2-isopropyl-5-methyl-9-methylene-	C_15_H_24_	204	13.863	2.46
	Sesquiterpene alcohol	τ-Muurolol	C_15_H_26_O	222	14.044	1.38
	Sesquiterpene	α-Calacorene	C_15_H_20_	200	14.194	0.29
	Sesquiterpene alcohol	1-((1S,3aR,4R,7S,7aS)-4-Hydroxy-7-isopropyl-4-methyloctahydro-1H-inden-1-yl) e	C_15_H_26_O_2_	238	15.061	14.67
	Sesquiterpene alcohol	(−)-Spathulenol	C_15_H_24_O	220	15.189	1.19
	Sesquiterpene	(3S,6S)-6-Isopropyl-3-methyl-2-(propan-2-ylidene)-3-vinylcyclohexanone	C_15_H_24_O	220	15.650	2.08
	Diterpenoid	Neophytadiene	C_20_H_38_	278	15.721	2.57
	Sesquiterpene	Isoaromadendrene epoxide	C_15_H_24_O	220	15.839	4.23
	Aldehyde	6-Nonenal, 3,7-dimethyl-	C_11_H_20_O	168	15.908	2.39
	Steroid	Pregn-4-ene-1,20-dione, 12-hydroxy-16,17-dimethyl-	C_23_H_34_O_3_	358	16.165	4.19
	Phthalate ester	Phthalic acid, 4,4-dimethylpent-2-yl isobutyl ester	C_19_H_28_O_4_	320	16.260	1.18
	Spiroketone	Spiro [2.5] octane, 5,5-dimethyl-4-(3-oxobutyl)-	C_14_H_24_O	208	16.677	0.74
	Acetic acid ester	Acetic acid, 3-(2,2-dimethyl-6-methylene-cyclohexylidene)-1-methyl-butyl ester	C_16_H_26_O_2_	250	16.876	1.40
	Spiroketone	Spiro [2.5] octane, 5,5-dimethyl-4-(3-oxobutyl)-	C_14_H_24_O	208	17.150	0.58
	Tricyclic diterpenoid	4,6,10,10-Tetramethyl-5-oxatricyclo [4.4.0.0(1,4)] dec-2-en-7-ol	C_13_H_20_O_2_	208	17.204	1.61
	Kaurane diterpenoid	Kauran-18-aL, 17-(acetyloxy)-, (4β)-	C_22_H_34_O_3_	346	17.764	1.94
	Triterpenoid	3-O-Acetyl-6-methoxy-cycloartenol	C_33_H_54_O_3_	498	18.289	2.54
	Diterpenoid	3,7,11,15-Tetramethyl-2-hexadecen-1-ol	C_20_H_40_O	296	18.634	2.95
	Alkane	Tridecane, 5-propyl-	C_16_H_34_	226	21.974	0.60
	Alkane	Hentriacontane	C_31_H_64_	436	23.800	11.61
	Triterpene	Squalene	C_30_H_50_	410	25.231	1.24
	Alkane	Pentatriacontane	C_35_H_72_	492	26.266	15.40
	Flavonoid	2-(3,4-Dimethoxyphenyl)-7-hydroxy-3-methoxy-4H-chromen-4-one	C_18_H_16_O_6_	328	28.975	2.36
	Alkane	Tetratetracontane	C_44_H_90_	618	29.893	17.17

In the ethyl acetate extract, 27 secondary metabolites were identified, including sesquiterpenoids, sesquiterpenes, diterpenoids, and flavonoids (Table 2). Tetratetracontane was the most abundant (17.17%) of the total peak area, followed by pentatriacontane (15.40%), and the sesquiterpene alcohol 1-((1S,3aR,4R,7S,7aS)-4-Hydroxy-7-isopropyl-4-methyloctahydro-1H-inden-1-yl) (14.67%) in the ethyl acetate leaf extract. α-Calacorene (0.29%), Spiro [2.5] octane (0.58%) and Ylangene (0.68%) were the least abundant in the ethyl acetate leaf extract of the study plant (Table 2). Notably, the ethyl acetate extract was rich in alkanes, such as Hentriacontane and Pentatriacontane, as well as various sesquiterpene alcohols and sesquiterpenes (Table 2).

### 3.2 *In vitro* cytotoxicity of the leaf extracts of *Vitex doniana*

#### 3.2.1 Cytotoxicity of the study extracts against Vero CCL-81 cells

**FIGURE 1:**
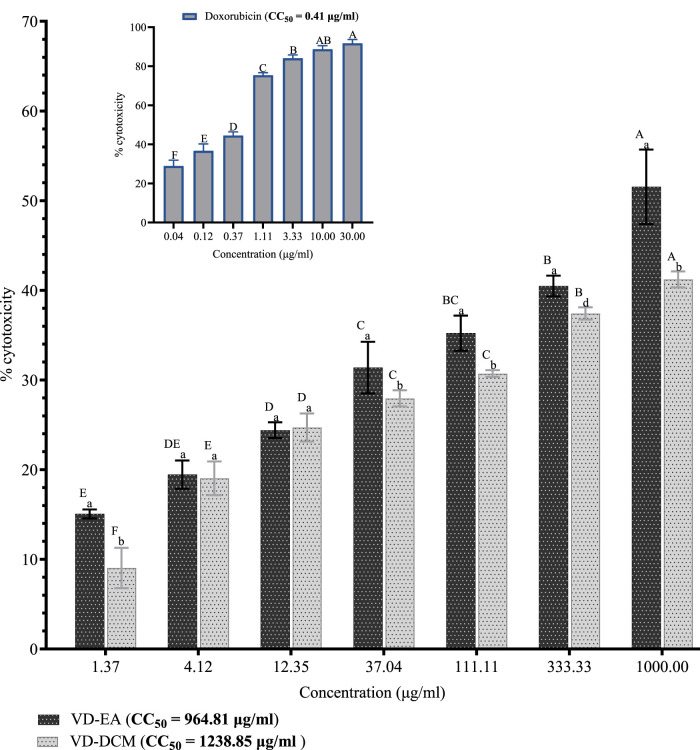
Cytotoxic effects of *V. doniana* leaf extracts against Vero CCL-81 cells. The results are presented as for four replicate experiments. Bars with different superscript uppercase letters across concentrations are significantly different (One-Way ANOVA with Tukey’s post hoc test), and those with different subscript lowercase letters within the same concentration are significantly different (*P*< 0.001; unpaired student t-test); VD-DCM: Dichloromethane leaf extract of V. doniana; VD-EA: Ethyl acetate leaf extract of *V. doniana*; CC_50_: Median cytotoxic concentration.

Generally, the results showed significant concentration-dependent increases in percentage cytotoxicity in Vero CCL-81 cells treated with the ethyl acetate and dichloromethane leaf extracts of *V. doniana* (*P*< 0.001; Figure 1). Comparatively, the ethyl acetate leaf extract of *V. doniana* exhibited significantly higher percentage cytotoxicity than the dichloromethane extract at a concentration of 1.37 μg/mL (*P*< 0.001; Figure 1). No significant difference was observed between the percentage cytotoxicity observed in cells treated with the ethyl acetate and dichloromethane extracts at concentrations of 4.12 μg/mL and 12.35 μg/mL (*P*> 0.001; Figure 1). However, at concentrations of 37.04 μg/mL, 111.11 μg/mL, 333.33 μg/mL, and 1,000 μg/mL, the dichloromethane leaf extract of *V. doniana* exhibited significantly higher cytotoxicity than the ethyl acetate extract (*P*< 0.001; Figure 1). Notably, Doxorubicin imparted significant cytotoxicity (*P*< 0.001) to Vero CCL-81 cells and assumed a concentration-dependent pattern, with a very low CC_50_ (0.41 μg/mL) as shown in Figure 1. Moreover, the dichloromethane leaf extract of *V. doniana* had a higher CC_50_ (1,238.85 μg/mL) compared to the ethyl acetate extract (CC_50_ = 964.81 μg/mL) (Figure 1).

#### 3.2.2 Effects of the leaf extracts of *Vitex doniana* on prostate cancer cells (DU-145)

**FIGURE 2:**
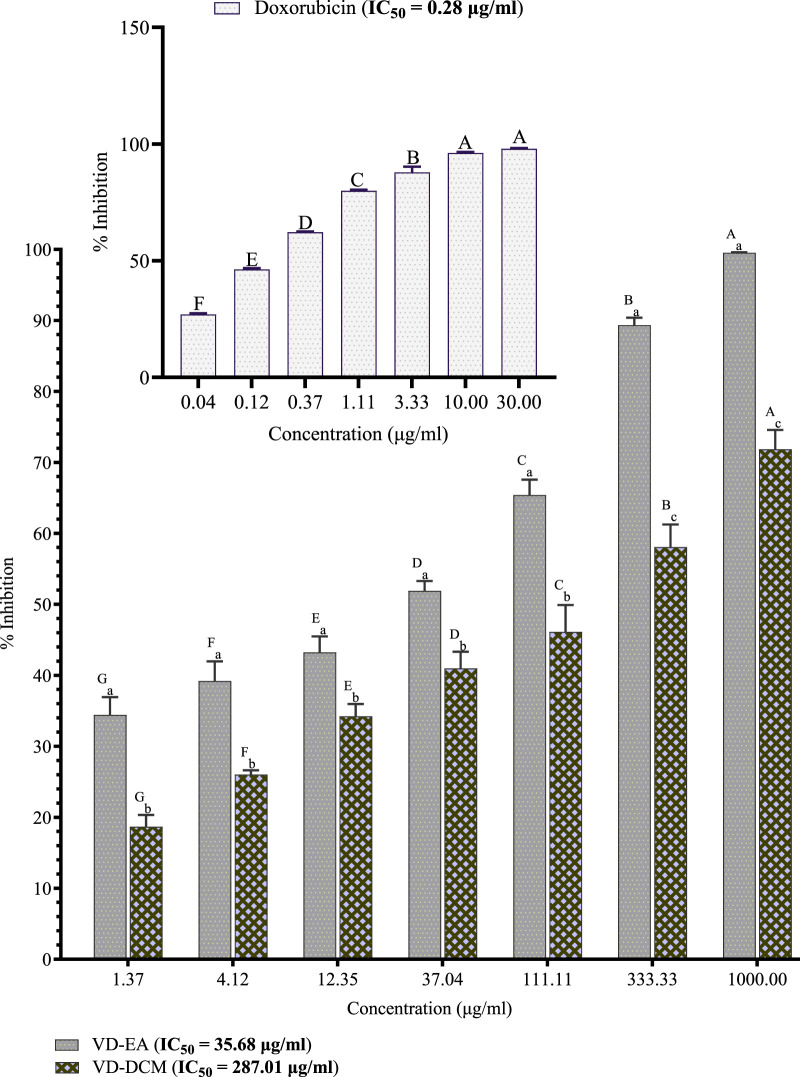
Anti-prostate cancer effects of *Vitex doniana* leaf extracts against DU-145 cells. The results are presented as for four replicate experiments. Bars with different superscript uppercase letters across concentrations are significantly different (One-Way ANOVA with Tukey’s post hoc test), and those with different subscript lowercase letters within the same concentration are significantly different (*P*< 0.001; unpaired student t-test); VD-DCM: Dichloromethane leaf extract of *V. doniana*; VD-EA: Ethyl acetate leaf extract of V. doniana; IC_50_: Median cytotoxic concentration.

This study also investigated the cytotoxic effects of the ethyl acetate and dichloromethane leaf extracts of *V. doniana* on prostate cancer cells (DU-145). The results showed that the percentage inhibitions of DU-145 cell growth by the ethyl acetate leaf extracts of *V. doniana* increased significantly with increasing concentration (*P*< 0.001; Figure 2). Notably, the ethyl acetate extract of this plant exerted significantly higher inhibitions of DU-145 cells’ growth than the dichloromethane extract at all the studied concentrations (*P*< 0.001; Figure 2). Moreover, this study revealed that the IC_50_ value of the ethyl acetate leaf extract of *V. doniana* was lower than that of the dichloromethane extract against DU-145 cells, and both were higher than that of doxorubicin (Figure 2).

#### 3.2.3 Selectivity indices

The selectivity indices (SI) of each studied plant extracts were computed to determine their ability to exert cytotoxic effects on cancer cells selectively while sparing normal cells. The results showed that the ethyl acetate leaf extract of *V. doniana* was relatively higher than that of the dichloromethane extract (Table 3).

**TABLE 3:** Selectivity indices of the studied plant extracts against prostate cancer Cells (DU-145).

Treatment	SI
VD-EA	27.04
VD-DCM	4.32
DOX	1.46

VD-EA: Ethyl acetate leaf extract of *Vitex doniana*; VD-DCM: Dichloromethane leaf extract of *Vitex doniana*; DOX: doxorubicin; SI: selectivity index.

### 3.3 Expression levels of selected genes in DU-145 cells treated with the plant extracts

**FIGURE 3:**
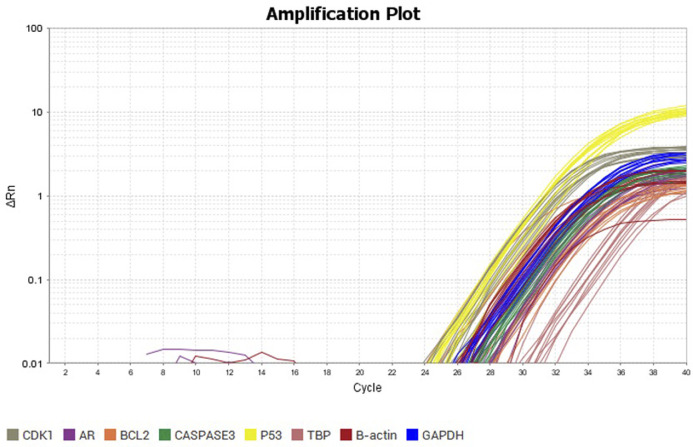
RT-qPCR amplification plot of the selected genes in DU-145 cells treated with the dichloromethane and ethyl acetate leaf extracts of *V. doniana*.

The expression profile of the selected genes in DU-145 cells treated with the plant extracts was determined in this study. The RT-qPCR output is shown in Figure 3 and the results are summarised in Table 4. The results showed that the expression of the *ar* gene in DU-145 cells treated with the ethyl acetate leaf extract of *V. doniana* was significantly higher than in similar cells treated with the dichloromethane extract of the same plant (*P*< 0.001; Table 4). It was also observed that the expression of the *bcl*_*2*_ gene in DU-145 cells treated with the dichloromethane leaf extract of *V. doniana* was significantly higher than in cells treated with the ethyl acetate extract and the control (*P*< 0.001; Table 4).

**TABLE 4:** Target gene expression profile in DU-145 cells treated with the selected plant extracts.

Treatment	RQ values of target genes				
	*Ar*	*bcl*_*2*_	*caspase 3*	*cdk-1*	*p*53
VD-DCM	1.23 ± 0.00^b^	3.10 ± 0.00^a^	0.95 ± 0.00^b^	0.63 ± 0.000^c^	0.76 ± 0.00^c^
VD-EA	1.50 ± 0.00^a^	1.09 ± 0.00^b^	0.79 ± 0.00^c^	0.86 ± 0.000^b^	0.92 ± 0.00^b^
Ctrl	1.00 ± 0.00^c^	1.00 ± 0.00^c^	1.00 ± 0.00^a^	1.00 ± 0.000^a^	1.00 ± 0.00^a^

The results are presented as $\bar{x}\pm SD$ for three replicate experiments. Means with different superscript letters within the same column are significantly different (*P*< 0.001; One-Way ANOVA with Tukey’s *post hoc* test); VD-DCM: Dichloromethane leaf extract of *Vitex doniana* (287.01 μg/mL); VD-EA: Ethyl acetate leaf extract of *Vitex doniana* (38.68 μg/mL); Ctrl: Control/Untreated; *ar*: Androgen receptor gene; *bcl*_*2*_: B-cell lymphoma 2 gene; *caspase 3*: Cysteine-aspartic acid protease gene; *cdk-1*: Cyclin-dependent Kinase 1/Cell division cycle protein 2 gene; *p*53: Tumour Protein 53 gene.

The results showed a significantly higher expression of the *caspase-3* gene in DU-145 cells treated with the dichloromethane leaf extract of *V. doniana* (*P*< 0.001); however, these expression levels were significantly lower than that observed in the control cells (*P*< 0.001; Table 4). Besides, the expression levels of *cdk-1* and *p53* genes in DU-145 cells treated with the ethyl acetate leaf extract of *V. doniana* were significantly higher than those observed in cells treated with the dichloromethane extract (*P*< 0.001); however, these expression levels were significantly lower than that observed in control cells (*P*< 0.001; Table 4).

## 4 Discussion

Despite significant advancements in modern medicine, anticancer therapy continues to face considerable hurdles, including prohibitive costs, limited availability, resistance to chemotherapy, and the severe side effects, often resulting in detrimental long-term consequences (Zugazagoitia et al., 2016). These challenges are particularly pronounced in less-developed regions, such as Sub-Saharan Africa, where inadequate healthcare infrastructure exacerbates the cancer burden (Sung et al., 2021). Considering these issues, there is an urgent need for alternative approaches that focus on identifying, optimising, and developing efficacious, safe, and cost-effective treatments, particularly those derived from botanical sources. Accordingly, this study investigated the cytotoxicity and anti-prostate cancer potential of the studied extracts as potential sources of safe and efficacious chemotherapeutic lead molecules for drug development.

This study employed Gas Chromatography-Mass Spectrometry (GC-MS) to investigate the secondary metabolite composition of the studied *V. doniana* leaf extracts. The dichloromethane extract contained ten metabolites, with γ-Sitosterol emerging as the most abundant. γ-Sitosterol possesses considerable anti-inflammatory, hypolipidemic, and anticancer properties, which it exerts by inducing apoptosis and inhibiting cancer cell proliferation (Ahamed et al., 2022). The presence of other bioactive secondary metabolites such as stigmasta-3,5-dien-7-one and Stigmast-4-en-3-one, known for their antioxidant and anti-inflammatory capabilities, further supports the medicinal potential of the plant for developing anticancer agents (Bakrim et al., 2022). Furthermore, lup-20(29)-en-3-one detected in the dichloromethane leaf extract of *V. doniana* has been reported to exhibit anti-cancer activity towards different types of cancer cells lines, including breast cancer (MCF7), colon carcinoma (HCT116), human lung adenocarcinoma (A549), and prostate cancer (PC3) by promoting apoptosis, modulating the cell cycle regulatory proteins (Yan et al., 2018). Besides, lup-20(29)-en-3-one can prevent tumour angiogenesis and metastasis through its effects on the vascular endothelial growth factor (VEGF) and epithelial-mesenchymal transition (EMT) signalling pathways (Tsepaeva et al., 2017; Liu et al., 2017). Undoubtedly, the antiprostate cancer effects exhibited by this extract in the present study were attributed to these secondary metabolites, whose mechanism of action is believed to be similar to those reported previously (Ahamed et al., 2022; Bakrim et al., 2022; Yan et al., 2018).

The ethyl acetate leaf extract of *V. doniana* revealed 27 secondary metabolites, including sesquiterpenoids, diterpenoids, aldehydes, phthalate esters, and flavonoids. Research has shown that sesquiterpene alcohols could possibly inhibit the growth and spread of cancer cells by aiming at and inhibiting important tumour-promoting signals in the cell cycle and apoptotic cascade that participate in tumour advancement (do Nascimento et al., 2018). This study identified notable sesquiterpene alcohol, especially 1-((1S,3aR,4R,7S,7aS)-4-Hydroxy-7-isopropyl-4-methyloctahydro-1H-inden-1-yl), e τ-muurolol, and (−)-spathulenol in the ethyl acetate leaf extract of *V. doniana*, recognised for their anti-inflammatory and anticancer properties (do Nascimento et al., 2018). These metabolites potentially exert their anticancer effects by disrupting critical signalling pathways, including cell cycle regulation and apoptosis, associated with prostate cancer tumour development and progression (Madriwala et al., 2022). The broad spectrum of bioactivities exhibited by the secondary metabolites of the ethyl acetate leaf extract of *V. doniana*, especially the terpenoids, sesquiterpenes, and diterpenoids, highlight the potential to contribute to cancer treatment (Mandour et al., 2023).

The contrasting profiles of metabolites observed between the dichloromethane and ethyl acetate extracts can be attributed to the varying polarities of the solvents, which influence the types and quantities of metabolites extracted (Truong et al., 2019). These differences suggest that optimising extraction parameters, such as solvent polarity and method efficiency, could enhance the quality and quantity of secondary metabolites isolated from *V. doniana* and other medicinal plants (Truong et al., 2019). However, translating the promising anticancer potential of these secondary metabolites into clinical applications remains a significant challenge due to issues with standardisation, bioavailability, and regulatory hurdles (Arora et al., 2019). Nonetheless, with continued research and advances in extraction and analytical techniques, the pharmacological promise of *V. doniana* could pave the way for the discovery and development of novel anticancer therapies.

The *in vitro* cytotoxicity of the studied plant extracts was examined on both normal mammalian cells (Vero CCL-81) and cancerous cell line (DU-145) using the well-established MTT assay technique (Markossian et al., 2021). This method is renowned for its high sensitivity, reliability, and reproducibility, making it a widely used approach in assessing cell viability for drug toxicity and anticancer efficacy studies (Ghasemi et al., 2021). The assay leverages the activity of mitochondrial NAD(P)H-dependent cellular oxidoreductases, especially succinate dehydrogenase, which reduces MTT dye into a water-insoluble formazan complex. This formazan’s absorbance is measured at 570 nm after dissolution in dimethyl sulphoxide, providing a quantitative assessment of cellular metabolic activity, thereby allowing for the discernment of cytotoxic or cytostatic effects with high accuracy (Ghasemi et al., 2021).

In this study, the cytotoxicity of the plant extracts exhibited a dose-dependent effect, with higher concentrations correlating to increased toxicity towards Vero CCL-81 cells and a marked inhibition of proliferation of DU-145 cells. These findings align with previous research demonstrating a similar concentration-dependent increase in cytotoxicity and growth inhibition, attributed to the presence of specific secondary metabolites, such as γ-sitosterol, stigmasta-3,5-dien-7-one, and stigmast-4-en-3-one (Muruthi et al., 2023). The cytotoxic and antiproliferative actions of these secondary metabolites are thought to occur through mechanisms such as inhibiting cellular efflux at high concentrations (Moyo and Mukanganyama, 2015) and modulating receptor-mediated intracellular signalling pathways, including suppression of endoplasmic reticulum signalling (Almanza et al., 2019). Consistent with previous studies, the dichloromethane and ethyl acetate leaf extracts of *V. doniana* were found to be non-toxic to normal cells, further substantiating their anti-prostate cancer potential. Notably, the presence of crucial sesquiterpenes, such as τ-muurolol and (−)-spathulenol in the ethyl acetate extract and diterpenes like Neophytadiene, triterpenoids like lup-20(29)-en-3-one as well as phytosterols like Stigmasta-3,5-dien-7-one and stigmast-4-en-3-one in the dichloromethane extract target specific pathways in prostatic cancer, such as averting oxidative stress, reversing inflammation, triggering DNA repair mechanisms, among others, thereby halting its initiation and development (da Silva et al., 2019).

Cancer development in somatic cells is driven by a dynamic and multifaceted accumulation of genetic mutations rather than a singular defect (Somarelli et al., 2020). These mutations trigger uncontrolled cellular proliferation, leading to tumour growth, morphological aberrations, and malignancy due to the disruption of essential genes that regulate the cell cycle, proto-oncogenes, and tumour-suppressor genes (Roszkowska et al., 2022). This deregulation of cellular checkpoints promotes unchecked mutation accumulation, characterized by autonomous growth signalling, resistance to growth inhibitors, evasion of apoptosis, limitless replication potential, angiogenesis, tissue invasion, and metastasis (Ramachandran and Dörk, 2021). The present study aimed to evaluate the expression profiles of key cancer-related genes to assess the efficacy of selected plant extracts, considering their high selectivity indices against DU-145 cells and to elucidate their probable molecular mechanisms of action.

In prostate cancer (PCa) and other cancers linked to sex hormones, the androgen receptor (AR) plays a pivotal role in tumour progression (Eisermann and Fraizer, 2017). Upon ligand binding, AR translocates to the nucleus, dimerises, and binds to androgen response elements (ARE) on target genes, initiating transcriptional activation by recruiting transcriptional machinery (Fujita and Nonomura, 2019). Pharmacological strategies to inhibit AR function often focus on disrupting ligand binding or interactions with coregulatory proteins to impede disease progression (Shah et al., 2020). Nevertheless, resistance to AR-targeting therapies remains a significant challenge in PCa management, often driven by reactivation of the AR axis via amplification, mutations, or the expression of AR variants (ARVs) that maintain constitutive activity independent of androgens, as observed in castration-resistant prostate cancer (Messner et al., 2020). Notably, treatment of DU-145 cells with the dichloromethane and ethyl acetate leaf extracts from *V. doniana* demonstrated limited influence on AR signalling, suggesting that the antiproliferative effects of these extracts may be mediated through an intricate modulation of other genes’ expression profiles.

Research indicates that *bcl-2* plays a critical role in inhibiting apoptosis by preventing cytochrome c release and impeding the activation of caspases, which are essential for cell death (Warren et al., 2019). This overexpression of *bcl-2* is a hallmark of various cancers, promoting their progression by initiating angiogenesis and enhancing cell survival (Pisani et al., 2020). Consequently, targeting the *bcl-2* gene and its protein product through chemotherapeutic agents has emerged as a pivotal cancer treatment strategy (Qian et al., 2022). Plant extracts that inhibit *bcl-2* gene overexpression hold significant potential as anticancer agents or lead compounds in drug development, given their regulatory influence on cell growth, proliferation, cell cycle, DNA repair, and tumour development (Zhang et al., 2021). In this study, DU-145 cells treated with the dichloromethane leaf extract of *V. doniana* exhibited significantly higher *bcl-2* expression than those treated with the ethyl acetate extract of this plant or control cells, underscoring the apoptotic potential of these extracts. These results align with previous research that has demonstrated reduced *bcl-2* expression in cancer cells treated with plant extracts, reinforcing their potential to induce apoptosis (Haselager et al., 2020).

*Caspase-3* plays a multifaceted role in tumour cell death mechanisms, exhibiting distinct effects compared to other caspases (Kostova et al., 2021). The intrinsic apoptotic pathway is initiated by mitochondrial damage, resulting in the release of cytochrome c, which forms an apoptosome with *Apaf*-1 and *procaspase*-9, thereby activating *caspase*-9 (Silva et al., 2022). This activation subsequently cleaves and activates pro-*Caspase-3*/7, leading to cell death via cleavage of endogenous substrates (Boice and Bouchier-Hayes, 2020). In contrast, the extrinsic pathway is triggered by tumour necrosis factor-α (TNF-α) binding to death receptors on the cell surface, leading to the activation of caspase-8, which directly cleaves pro-*Caspase-3*, inducing apoptosis (Kostova et al., 2021). In this study, a normal expression of the *Caspase-3* gene was observed in prostate cancer cells (DU-145) treated with dichloromethane and ethyl acetate leaf extracts of *V. doniana*, suggesting the presence of secondary metabolites that modulate *Caspase-3* expression and activity, to execute apoptosis (Pisani et al., 2020). However, research indicate that *Caspase-3* overexpression may correlate with reduced survival rates in cancer patients and may promote tumour regrowth, chemotherapy resistance, and other detrimental effects. Thus, *Caspase-3* appears to exhibit a paradoxical role in tumour development and progression, necessitating further research to elucidate the complex dynamics between pro*Caspase-3* and *Caspase-3* in cancer (Eskandari and Eaves, 2022).

Cyclin-dependent kinases (CDKs), a family of serine/threonine kinases, play essential roles in controlling both cell division and transcription in response to external and internal signals, with their activation dependent on complex formation with cyclins (Łukasik et al., 2021). The *cdk*1’s centrality to the G2/M and G1/S phase transitions renders it indispensable for cell cycle progression, with its dysregulation frequently driving oncogenesis (Sofi et al., 2022). Interestingly, analysis of The Cancer Genome Atlas (TCGA) data highlights *cdk1*’s overexpression in malignancies (Chandrashekar et al., 2022). Notably, treatment with dichloromethane and ethyl acetate extracts of *V. doniana* normalised *cdk*1 expression in DU-145 cancer cells, suggesting these extracts may restore *cdk*1’s function and mitigate prostate cancer progression (Wang et al., 2023).

Acting as a transcription factor, *p53* responds to various cellular stress signals—such as oncogene activation, DNA damage, and replication stress—by undergoing post-translational modifications that influence specific gene transcription, dictating cellular outcomes (Voskarides and Giannopoulou, 2023). Recent research has demonstrated *p53*’s role in pathways involving autophagy, cell metabolism, ferroptosis, and reactive oxygen species production (Hernández Borrero and El-Deiry, 2021). Its mutation is evinced in advanced prostate cancer, where germline and somatic mutations in DNA damage repair genes are prevalent (Roszkowska et al., 2022). In this study, we investigated the expression of the *p53* gene in prostate cancer (DU-145) cells treated with plant extracts. Notably, the relatively normal *p53* expression observed in cells treated with dichloromethane and ethyl acetate extracts from *V. doniana* suggests that their secondary metabolites such as phytosterols, triterpenes, sesquiterpenes, and flavonoids probably modulated its expression to drive apoptosis and ultimately avert cancer development and progression (Tsepaeva et al., 2017; Liu et al., 2017; Mandour et al., 2023).

Therefore, considering the longstanding usage of this plant in traditional medicine to treat prostate cancer among other conditions, this study, for the first time, provides a partial validation of its medicinal potential. Besides, the findings reported herein lay a basis for further empirical studies aimed at establishing its *in vivo* safety and efficacy, potentially leading to the discovery of botanical-based antiprostate cancer drugs.

## 5 Conclusions and recommendations

Based on the study findings, the dichloromethane and ethyl acetate leaf extracts of *V*. *doniana* contain bioactive secondary metabolites with antioxidant, anti-inflammatory, and anticancer properties, positioning them as potential sources of lead compounds for managing oxidative stress-related diseases, especially prostate cancer. These extracts exhibit selective cytotoxicity against prostate cancer cells (DU-145) while sparing normal mammalian cells (Vero CCL-81), underscoring their antiprostate cancer potential. Moreover, these extracts modulated the expression of key genes involved in cancer initiation and progression, providing an insight into their probable molecular mechanism of action. Future research should focus on isolating and characterising these secondary metabolites, assessing their *in vivo* efficacy and safety, and clarifying their precise mechanisms of action. Additionally, investigations into other extracts of *V. doniana* and their combinations, as well as translational studies to facilitate clinical application, are recommended.

## Data Availability

All the data and materials used in this study are included in the article. Additional information may be provided by the corresponding author(s) upon reasonable request.
